# Integrated single-cell and bulk tissue analyses reveal distinct macrophage subtypes and a candidate prognostic signature in colorectal cancer: implications for tumor immune characterization

**DOI:** 10.3389/fimmu.2026.1902264

**Published:** 2026-09-18

**Authors:** Hui Chen, Zhipeng Li, Zhen Lin, Lijun Wan, Yuhang Gong, Zhibin Lv, Jinfeng Hu, Dun Pan

**Affiliations:** 1 Department of Gastrointestinal Surgery, First Affiliated Hospital of Fujian Medical University, Fuzhou, China; 2 Fujian Research Institute of Abdominal Surgery, Fuzhou, China; 3 First Affiliated Hospital of Fujian Medical University Binhai Campus, Fuzhou, China; 4 Fujian Medical University School of Basic Medical Sciences, Fuzhou, China

**Keywords:** CCL24, colorectal cancer, macrophage, MMP12, prognosis, single-cell RNA sequencing, tumor immunology

## Abstract

**Introduction:**

Colorectal cancer (CRC) represents a major global health burden, marked by high morbidity and mortality rates that place a considerable strain on healthcare systems.

**Methods:**

This study leveraged integrated bioinformatic analyses, single-cell RNA sequencing, and clinical sample validation to investigate the role of macrophage-related genes (MRGs) in CRC, with the goal of deepening our understanding of the complex interplay within the tumor microenvironment.

**Results:**

Differential expression analysis comparing CRC tumor and normal tissues in the TCGA-COADREAD cohort identified 1,962 differentially expressed genes (DEGs). In parallel, a predefined set of 1,719 MRGs was curated from public databases and the literature to define the macrophage-related biological context. By integrating the bulk transcriptomic DEGs, single-cell macrophage subtype-specific genes, and the predefined MRG set, we identified eight hub macrophage-related DEGs (MRDEGs) implicated in CRC. Functional enrichment analysis of these eight MRDEGs revealed significant roles in immune-regulatory processes, including leukocyte chemotaxis, eosinophil chemotaxis, chemokine receptor binding, and the chemokine signaling pathway. From these eight hub MRDEGs, we selected *CCL24* and *MMP12* via LASSO-Cox regression to construct a prognostic risk model. Immune infiltration analysis using CIBERSORT revealed significant differences (*p* < 0.05) in the abundance of nine immune cell types (including M0/M2 Macrophages, CD8+ T cells, T follicular helper cells, regulatory T cells (Tregs), memory B cells, monocytes, eosinophils, and neutrophils) between the risk groups defined by this macrophage-related signature. Drug sensitivity analysis further revealed that the high-risk group was significantly less responsive to sorafenib (*p* < 0.05). The model provides candidate molecular clues for further investigation of the CRC immune microenvironment and prognostic stratification related to macrophage biology. We further performed experimental validation of the differentially expressed genes using clinical CRC samples and assessed their expression at both the transcriptional and protein levels.

**Discussion:**

This study provides a candidate prognostic stratification model that requires further validation in independent cohorts and prospective studies, offering a macrophage-related prognostic clue for future investigations.

## 1 Introduction

Worldwide, colorectal cancer (CRC) ranks third in incidence and second in cancer-related mortality, accounting for approximately 2 million new cases and over 900,000 deaths annually, thereby posing a severe burden on public health (1). The disease not only causes significant physical and psychological distress to patients, but also imposes a substantial burden on healthcare systems and socioeconomic resources. Despite considerable progress having been made in recent years in treatment modalities (2)—including surgical resection, chemotherapy, targeted therapy, and immunotherapy—several challenges remain. These include a relatively low early diagnosis rate (3), the development of drug resistance (4), and the limited proportion of patients who respond to immunotherapy (5). All of these factors constrain further improvement in patient outcomes. Therefore, further investigation into the pathogenesis and molecular characteristics of CRC, particularly focusing on macrophage-related genes, is crucial for the development of novel targeted therapies.

Recent studies have revealed that immune cells within the tumor microenvironment, particularly macrophages, are closely associated with tumor progression and treatment response (6). Macrophages are pivotal in establishing an immunosuppressive microenvironment (7). This environment not only directly supports cancer cell survival, progression, invasion, and metastasis but also promotes angiogenesis and facilitates drug resistance (8, 9). Despite the growing recognition of macrophage importance, existing CRC prognostic models are predominantly constructed based on bulk transcriptomic differential expression (10) or immune infiltration scores (11). These models often lack the granularity to trace the candidate genes back to their specific cellular origins and functional subtypes within the tumor microenvironment, particularly at the single-cell level (12). Therefore, this study was designed to address this gap by integrating single-cell RNA sequencing to define macrophage heterogeneity, multi-cohort bulk transcriptomic analysis to identify robustly dysregulated genes, and LASSO-Cox regression to construct a candidate prognostic signature with clear macrophage-related biological context. These bioinformatic predictions were preliminarily validated using clinical CRC samples.

## 2 Materials and methods

### 2.1 Data acquisition and preprocessing

Transcriptomic data and corresponding clinical information (**Table 1**) for the TCGA-COADREAD CRC cohort were retrieved from The Cancer Genome Atlas (TCGA) and the UCSC Xena database (13). The TCGA-COAD (colon adenocarcinoma) and TCGA-READ (rectum adenocarcinoma) datasets were combined as TCGA-COADREAD, as they belong to the same colorectal cancer spectrum and are commonly analyzed together in TCGA-based CRC transcriptomic and prognostic studies. This dataset served as the training set. Additionally, four bulk RNA-seq datasets (GSE20916 (14), GSE73360 (15), GSE44861 (16), and GSE74602 (17)) and one single-cell RNA-seq (scRNA-seq) dataset (GSE166555) (18) were obtained from the Gene Expression Omnibus (GEO) and served as validation sets. All datasets were derived from Homo sapiens colon tissue. Briefly, GSE20916 (platform GPL570) included 81 CRC and 24 control samples; GSE73360 (platform GPL17586) included 55 CRC and 31 control samples; GSE44861 (platform GPL3921) included 56 CRC and 55 control samples; and GSE74602 (platform GPL6104) included 30 CRC and 30 control samples. The independence of these GEO datasets was verified by checking GEO accession numbers, platforms, sample IDs, and source publications; no significant sample overlap was identified. All bulk RNA-seq data were normalized and processed using the limma R package. A comprehensive list of 1,719 MRGs was curated by integrating searches from the GeneCards and MSigDB databases (19), as well as relevant literature (20) (**Supplementary Table 1**). This pre-defined MRGs set (**Supplementary Table 1**) was subsequently used as the input for the AUCell algorithm to score gene set activity across cells.

**Table 1:** Patient Characteristics of CRC patients in the TCGA-COADREAD datasets.

Characteristics	Overall
Pathologic T stage, n (%)	
T1	20 (3.1%)
T2	111 (17.3%)
T3	436 (68%)
T4	74 (11.5%)
Pathologic N stage, n (%)	
N0	368 (57.5%)
N1	153 (23.9%)
N2	119 (18.6%)
Pathologic M stage, n (%)	
M0	475 (84.2%)
M1	89 (15.8%)
Gender, n (%)	
Female	301 (46.7%)
Male	343 (53.3%)
Age, n (%)	
<= 65	276 (42.9%)
> 65	368 (57.1%)

TCGA, The Cancer Genome Atlas; CRC, Colorectal Cancer.

### 2.2 Single-cell data processing and identification of key genes

The scRNA-seq dataset (GSE166555) was processed using the Seurat R package (version 4.0). A Seurat object was first constructed, and quality control (QC) was performed to retain cells meeting the following criteria: number of detected genes (nFeature_RNA) > 200, total UMI count (nCount_RNA) ≥ 200, and mitochondrial gene percentage (percent.mt) < 20%. The expression matrix was then normalized using the NormalizeData function, and the top 2,000 highly variable genes were identified using FindVariableFeatures. All genes were scaled with the ScaleData function, regressing out the percent.mt. Principal component analysis (PCA) was performed for dimensionality reduction, and the first 15 principal components were selected for downstream analyses, including graph-based clustering (FindNeighbors, FindClusters) and non-linear dimensionality reduction via Uniform Manifold Approximation and Projection (UMAP). The clustering resolution was set to 0.1. Cell type annotation was performed by combining automated annotation results from SingleR with canonical marker genes curated from the CellMarker 2.0 database. Of note, the initial graph-based clustering and the subsequent major-cell-type annotation operate at different resolution levels: clusters that are spatially separated in the UMAP embedding may still be assigned to the same major cell type if they share lineage-defining marker gene expression. For instance, two UMAP-separated clusters both positive for B-cell lineage markers (e.g., CD79A and MS4A1) were both annotated as B cells and are referred to as “B cell cluster 1” and “B cell cluster 2” to reflect their shared lineage identity and distinct transcriptional states. The major cell types identified included T cells, B cells, macrophages, platelets, neutrophils, erythrocytes, and neural progenitor cells.

Following initial clustering, the macrophage, epithelial cell, and fibroblast populations were extracted for further subclustering analysis. Each subcluster was subsequently annotated based on its most differentially expressed marker genes and inferred functional states. Differentially expressed genes (DEGs) were identified using cutoff of (|log_2_FC| > 3, adj. *p*-value < 0.0001). The intersection of these subtype-specific DEGs with the predefined MRGs yielded 56 macrophage-related DEGs (MRDEGs). Gene Set Variation Analysis (GSVA) (21) was performed on these cell subtypes to dissect pathway activities. SCENIC (22), CellPhoneDB (23), and pseudotemporal trajectory inference analyses were conducted to explore regulatory networks, cell-cell communication, and potential differentiation dynamics among these subtypes.

### 2.3 Bulk RNA-seq analysis and functional characterization

The limma package was employed for differential expression analysis comparing CRC and normal samples across all bulk RNA-seq datasets, with a cutoff of |log_2_FC| > 1 and an adjusted *p*-value < 0.05. To identify robust candidate genes, MRDEGs that were consistently dysregulated in at least three out of five bulk cohorts (TCGA-COADREAD, GSE20916, GSE73360, GSE44861, and GSE74602) were defined as hub genes. The differential expression profiles of all 56 MRDEGs across these five cohorts, including logFC, *P* value, adjusted *P* value, direction, and significance status, are provided in **Supplementary Table 2**. The clusterProfiler R package was applied to conduct Gene Ontology (GO) and Kyoto Encyclopedia of Genes and Genomes (KEGG) pathway enrichment analyses on the final set of MRDEGs. Additionally, the patterns of somatic mutations and copy number variations within the TCGA-CRC cohort were evaluated by maftools and subsequently visualized through ggplot2.

### 2.4 Construction and validation of a prognostic signature

To construct a prognostic risk score, we subjected the expression profiles of the final MRDEGs in the TCGA cohort to LASSO Cox regression analysis, employing 10-fold cross-validation, as implemented in the glmnet R package. The lambda.min value (the lambda that minimizes the cross-validated deviance) was used to build the final model, as lambda.1se (the largest lambda within one standard error of the minimum) resulted in no retained variables.


$$\text{Risk}Score=\underset{i}{\sum }Coefficient\;(gene_{i})*mRNA\;Expression\;(gene_{i})$$

Patients were then categorized into high-risk and low-risk groups based on the median risk score of the TCGA cohort: patients with a risk score higher than the median were defined as the high-risk group, and those with a risk score equal to or lower than the median were defined as the low-risk group. The continuous risk score was used for model construction and individual risk calculation, while the dichotomized high-/low-risk groups were used for group comparisons in risk distribution plots, immune infiltration analysis, drug sensitivity assessment, and immune checkpoint expression analysis. The independence and prognostic power of the signature were evaluated using univariate and multivariate Cox regression analyses, and a nomogram was developed (rms package). The model’s clinical utility was assessed using decision curve analysis (DCA) (24) and calibration curves.

### 2.5 Immune landscape and therapeutic response analysis

The immune cell infiltration profile of the TCGA cohort was quantified using single-sample gene-set enrichment analysis (ssGSEA) (25) and CIBERSORT (26). Consensus clustering (ConsensusClusterPlus) was applied to the ssGSEA matrix to define immune subtypes. Differences in Tumor Immune Dysfunction and Exclusion (TIDE) scores, tumor mutational burden (TMB), and microsatellite instability (MSI) among subtypes and risk groups were evaluated. Drug sensitivity (IC_50_) to common chemotherapeutic agents was predicted using the pRRophetic algorithm, and the expression profiles of immune checkpoint genes were compared between risk groups.

### 2.6 Clinical sample validation

Approval for this study was granted by the Ethics Committee of the First Affiliated Hospital of Fujian Medical University (Approval No.: MRCTA, ECFAH of FMU [2021]423). A total of pathologically confirmed CRC patients were enrolled. Paired CRC tumor tissues and adjacent normal tissues (at least 5 cm from tumor margin) were collected. Inclusion criteria were: (i) histopathologically confirmed CRC after surgical resection; (ii) no prior radiotherapy, chemotherapy, immunotherapy, or other antitumor treatment before surgery; (iii) tissue samples of adequate quality for molecular analyses. Exclusion criteria were: (i) presence of other concurrent malignancies; (ii) receipt of neoadjuvant therapy; (iii) incomplete clinical data; (iv) poor tissue preservation or sample quality. Demographic and clinicopathological characteristics of the cohort (age, sex, tumor location, TNM stage, pathological stage, differentiation, lymph node metastasis) are summarized in **Table 2**. This validation cohort was independently collected at our center and had no sample overlap with the TCGA-COADREAD or GEO discovery datasets.

**Table 2:** Clinicopathological characteristics of the clinical validation cohort (n = 7 for qRT-PCR; n = 3 for Western blot).

Patient ID	Age (years)	Sex	Tumor location	TNM stage (T/N/M)	Pathological stage	Differentiation	Lymph node metastasis	Protein validation (western blot)
CRC-01	62	Male	Right colon	T3/N1/M0	IIIA	Poor	Yes (1/17)	Yes
CRC-02	55	Female	Rectum	T3/N0/M0	IIA	Moderate	No (0/15)	Yes
CRC-03	70	Male	Left colon	T4a/N2/M1	IV	Poor	Yes (11/28)	Yes
CRC-04	48	Female	Right colon	T3/N0/M0	IIA	Moderate	No (0/16)	No
CRC-05	65	Male	Sigmoid colon	T3/N1/M0	IIIB	Moderate	Yes (2/14)	No
CRC-06	73	Female	Rectum	T4b/N2/M0	IIIB	Poor	Yes (5/20)	No
CRC-07	59	Male	Right colon	T4a/N0/M0	IIB	Well	No (0/18)	No

The mRNA expression levels of the eight macrophage-related hub MRDEGs (*CCL24*, *ADAMDEC1*, *CHI3L1*, *GZMB*, *CCL23*, *CD163L1*, *MMP12*, and *CDKN3*) were assessed by quantitative real-time PCR, and the protein expression levels of CCL24 and MMP12 (the two genes constituting the final prognostic signature) were detected by immunoblotting. Both procedures were carried out according to previously reported methods (27). The primer sequences employed are summarized in **Table 3**.

**Table 3:** qRT-PCR primer sequences.

Primers	Primer sequence(5′-3′)
CCL24	F: ACATCATCCCTACGGGCTCT R: CTTGGGGTCGCCACAGAAC
ADAMDEC1	F:GGCCTTGGTAGGTATGGAAATC R:CCCCAGGTTAGAACTGTGCC
CHI3L1	F:AAGCAACGATCACATCGACAC R:TCAGGTTGGGGTTCCTGTTCT
GZMB	F:CCCTGGGAAAACACTCACACA R:CCCTGGGAAAACACTCACACA
CCL23	F:CATCTCCTACACCCCACGAAG R:GGGTTGGCACAGAAACGTC
CD163L1	F:GCTGTGGTAACTTGCATCCTG R:GCAGTAGTGTTCCACCCATCA
MMP12	F:CATGAACCGTGAGGATGTTGA R:GCATGGGCTAGGATTCCACC
CDKN3	F:TCCGGGGCAATACAGACCAT R:GCAGCTAATTTGTCCCGAAACTC

All primers were synthesized by Shanghai Shenggong Bioengineering Co., Ltd. (F, forward; R, reverse).

### 2.7 Statistical analysis

All statistical analyses were performed using R software (version 4.3.2). Comparisons between groups were made using Student’s t-test, the Mann-Whitney U test, or the Kruskal-Wallis test, depending on the underlying assumptions. Correlation analyses were conducted based on Spearman’s method. A two-tailed *p*-value below 0.05 was regarded as indicating statistical significance. For multiple comparisons involving multiple immune cell types, immune checkpoints, and drug sensitivity analyses, the Benjamini-Hochberg (BH) method was applied to adjust *P* values, and an adjusted *P* value or false discovery rate (FDR) < 0.05 was used as the primary threshold for statistical significance. *P* < 0.05 was otherwise considered statistically significant for single comparisons.

## 3 Result

### 3.1 Single-cell profiling reveals macrophage heterogeneity and core candidate genes in colorectal cancer

To obtain a high-resolution view of the CRC tumor microenvironment and identify macrophage subtype-related genes as a starting point for subsequent bulk cohort screening and prognostic modeling, we analyzed the scRNA-seq dataset GSE166555. After quality control, we identified seven major cell populations, including epithelial cells, T cells, B cells, macrophages, and mast cells (**Figure 1A**). Notably, two spatially separated clusters in the UMAP embedding were annotated as B cells. Both clusters exhibited B-cell lineage marker expression (e.g., CD79A and MS4A1) and were therefore assigned to the B-cell lineage; however, they represented distinct transcriptional states. To reflect this, they are labeled as “B cell cluster 1” and “B cell cluster 2” in **Figure 1A**. To further support the major cell type annotation, we visualized the expression of representative marker genes for each major cell type on the UMAP embedding; the resulting feature plots are provided in **Supplementary Figure 1**. Scoring with the AUCell algorithm indicated that a pre-defined MRGs set (**Supplementary Table 1**) was most enriched within the macrophage cluster (**Figure 1B**), suggesting a higher activity of this gene set in macrophages, rather than indicating that these genes are exclusively expressed in macrophages. Further subclustering of macrophages revealed three distinct subtypes, which were characterized by distinct transcriptional signatures rather than a single marker gene. After carefully re-evaluating the correspondence among cluster identity, representative marker genes, and functional annotation, Cluster 0 was defined as inflammatory monocyte-like macrophages, characterized by high expression of FCN1, S100A8, S100A12, and VCAN, with CLC shown as the representative marker gene in the violin plot; Cluster 1 was defined as lipid-associated TAM-like macrophages, characterized by high expression of APOE, GPNMB, SELENOP, and FOLR2, with C2orf48 shown as the representative marker gene; and Cluster 2 was defined as proliferating macrophages, characterized by high expression of MKI67, TOP2A, TYMS, and UBE2C, with HS3ST2 shown as the representative marker gene (**Supplementary Figure 2A**). These subtypes were therefore named based on their multi-gene marker profiles and inferred functional states, rather than individual differentially expressed genes. To further illustrate the transcriptional distinctions among the three macrophage subtypes, we generated UMAP feature plots for the top five differentially expressed marker genes of each subtype, as shown in **Supplementary Figure 3**. To identify genes associated with macrophage heterogeneity, we performed differential expression analysis between these macrophage subtypes. The resulting differentially expressed genes, which are specific to macrophage subtypes, were then intersected with the predefined macrophage-related gene list. This intersection, rather than simply using all DEGs, yielded 56 MRDEGs (**Figure 1C**). GSVA indicated significant differences in pathway activities (**Table 4**), such as ribosome assembly and inflammatory response, among these subtypes (**Supplementary Figure 2B**). Novel subtype structures were also identified in epithelial cells and fibroblasts (**Supplementary Figures 4**, **S5**). These 56 MRDEGs served as a foundation for subsequent multi-cohort bulk screening to identify robustly dysregulated candidates.

**Figure 1:**
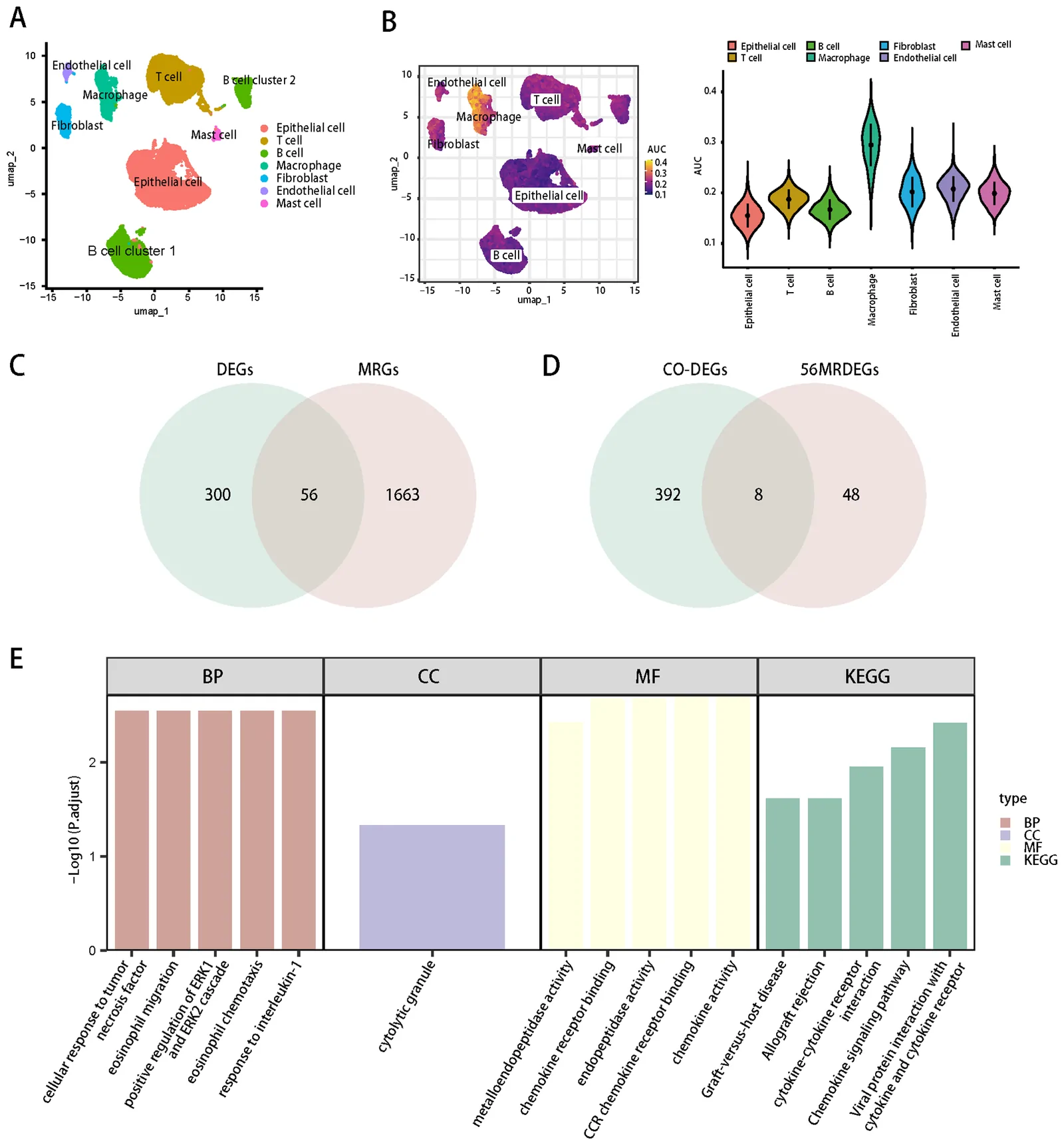
Single-cell transcriptomic landscape of CRC and identification of macrophage-related gene signatures. **(A)** UMAP plot of major cell types identified in the CRC scRNA-seq dataset (GSE166555). Cell types were annotated based on preliminary results from SingleR and further confirmed using canonical marker genes curated from the CellMarker 2.0 database. The major cell types identified include epithelial cells, T cells, B cells, macrophages, fibroblasts, endothelial cells, and mast cells. The two spatially separated B-cell-related clusters were both assigned to the B-cell lineage based on their B-cell lineage marker-gene profiles (e.g., CD79A and MS4A1) and are labeled as “B cell cluster 1” and “B cell cluster 2” in the figure to reflect their distinct transcriptional states. Representative marker-gene UMAP feature plots for these major cell types are provided in **Supplementary Figure 1**. **(B)** UMAP plot showing the AUCell activity score of the MRG set across all cells, confirming the highest activity in the macrophage cluster. **(C)** Enn diagram illustrating the intersection between subtype-specific DEGs and the pre-defined MRG set, yielding 56 MRDEGs. **(D)** Venn diagram showing the intersection between CO-DEGs (genes consistently dysregulated in at least three of five bulk transcriptomic cohorts) and 56 MRDEGs, yielding eight hub MRDEGs (*CCL24*, *ADAMDEC1*, *CHI3L1*, *GZMB*, *CCL23*, *CD163L1*, *MMP12*, and *CDKN3*). **(E)** Bar plot of the top enriched GO terms and KEGG pathways for the eight hub genes, highlighting their involvement in immune-related processes such as leukocyte chemotaxis and chemokine signaling pathway.

**Table 4:** Results of GSVA between macrophage cell subtype.

ID	logFC	AveExpr	t	P.Value	adj.P.Val	B
WP_CYTOPLASMIC_RIBOSOMAL_PROTEINS	0.951702	-0.601030	34.353231	7.99E-219	1.73E-218	487.920
KEGG_RIBOSOME	0.941190	-0.585818	32.927861	1.23E-203	2.59E-203	452.967
REACTOME_EUKARYOTIC_TRANSLATION_ELONGATION	0.933513	-0.574168	32.465750	8.91E-199	1.85E-198	441.785
WP_ACTIVATION_OF_NLRP3_INFLAMMASOME_BY_SARSCOV2	0.915609	-0.460603	20.883409	1.30E-90	2.08E-90	193.006
BILANGES_SERUM_AND_RAPAMYCIN_SENSITIVE_GENES	0.897378	-0.588745	36.234747	2.70E-239	6.07E-239	535.035
NIKOLSKY_BREAST_CANCER_19Q13.4_AMPLICON	0.896584	-0.896547	3942.266106	0.00E+00	0.00E+00	12945.754
REACTOME_RESPONSE_OF_EIF2AK4_GCN2_TO_AMINO_ACID_DEFICIENCY	0.887413	-0.552590	33.039762	8.11E-205	1.71E-204	455.686
REACTOME_CLEC7A_INFLAMMASOME_PATHWAY	0.885121	-0.396119	20.919388	6.72E-91	1.08E-90	193.664
MYLLYKANGAS_AMPLIFICATION_HOT_SPOT_30	0.880205	-0.410551	24.878119	1.64E-124	2.92E-124	270.937
REACTOME_DEX_H_BOX_HELICASES_ACTIVATE_TYPE_I_IFN_AND_INFLAMMATORY_CYTOKINES_PRODUCTION	0.876243	-0.663953	33.210510	1.27E-206	2.68E-206	459.844
REN_ALVEOLAR_RHABDOMYOSARCOMA_UP	0.047371	-0.070968	3.385266	7.20E-04	7.66E-04	-5.110
REACTOME_RESOLUTION_OF_D_LOOP_STRUCTURES	0.046168	-0.197831	3.306811	9.55E-04	1.01E-03	-5.371
WP_BASE_EXCISION_REPAIR	0.046010	-0.359748	2.921656	3.51E-03	3.69E-03	-6.567
KEGG_BASE_EXCISION_REPAIR	0.045489	-0.347148	2.885522	3.94E-03	4.14E-03	-6.672
HOLLERN_SOLID_NODULAR_BREAST_TUMOR_DN	0.045129	-0.111909	2.725233	6.46E-03	6.78E-03	-7.121
EBAUER_MYOGENIC_TARGETS_OF_PAX3_FOXO1_FUSION	0.043914	0.044395	2.131794	3.31E-02	3.44E-02	-8.559
REACTOME_INTRAFLAGELLAR_TRANSPORT	0.042029	-0.228637	2.738299	6.21E-03	6.52E-03	-7.085
SENESE_HDAC1_AND_HDAC2_TARGETS_DN	0.037946	-0.102330	3.427113	6.18E-04	6.58E-04	-4.968
MORI_PRE_BI_LYMPHOCYTE_UP	0.032234	-0.396592	3.830979	1.30E-04	1.40E-04	-3.508
BASAKI_YBX1_TARGETS_UP	0.023624	-0.303261	3.707647	2.13E-04	2.28E-04	-3.971

GSVA, Gene Set Variation Analysis.

Based on the TCGA-COADREAD cohort, differential expression analysis between tumor and normal tissues identified a total of 1,962 differentially expressed genes (DEGs), comprising 809 upregulated and 1,153 downregulated genes. This set of DEGs served as the foundation for subsequent cross-cohort integration (**Supplementary Table 2**). To further identify the most CRC-relevant core genes, the 56 MRDEGs were intersected with genes consistently dysregulated in at least three of the five independent bulk transcriptomic cohorts (CO-DEGs). This integrative approach yielded eight hub genes: *CCL24*, *ADAMDEC1*, *CHI3L1*, *GZMB*, *CCL23*, *CD163L1*, *MMP12*, and *CDKN3* (**Figure 1D**, Venn diagram). The detailed differential expression results for all 56 MRDEGs across each of the five bulk cohorts, which formed the basis for this selection, are provided in **Supplementary Table 3**. Their consistent differential expression between tumor and normal tissues was validated across all cohorts (**Supplementary Figure 6**). Functional enrichment analysis revealed these hub genes were significantly involved in immune-regulatory processes such as leukocyte chemotaxis, eosinophil chemotaxis, chemokine receptor binding, and chemokine signaling pathway (**Figure 1E**) (**Table 5**). The genomic alteration landscape of these eight genes is summarized in **Supplementary Figure 7**. These 8 hub genes, which retain both macrophage subtype-related information and stable differential expression in bulk CRC tissues, served as the foundation for subsequent analyses of tumor immune subtypes classification and prognostic modeling construction.

**Table 5:** Results of GO and KEGG enrichment analysis for MRDEGs.

ONTOLOGY	ID	Description	GeneRatio	BgRatio	pvalue	p.adjust	qvalue
BP	GO:0070555	response to interleukin-1	3/7	140/18870	1.37E-05	2.86E-03	1.18E-03
BP	GO:0048245	eosinophil chemotaxis	2/7	28/18870	4.44E-05	2.86E-03	1.18E-03
BP	GO:0070374	positive regulation of ERK1 and ERK2 cascade	3/7	218/18870	5.14E-05	2.86E-03	1.18E-03
BP	GO:0072677	eosinophil migration	2/7	32/18870	5.82E-05	2.86E-03	1.18E-03
BP	GO:0071356	cellular response to tumor necrosis factor	3/7	238/18870	6.68E-05	2.86E-03	1.18E-03
CC	GO:0044194	cytolytic granule	1/8	13/19886	5.22E-03	4.70E-02	3.30E-02
MF	GO:0008009	chemokine activity	2/7	49/18496	1.43E-04	1.94E-03	6.28E-04
MF	GO:0048020	CCR chemokine receptor binding	2/7	50/18496	1.49E-04	1.94E-03	6.28E-04
MF	GO:0004175	endopeptidase activity	3/7	389/18496	3.03E-04	2.13E-03	6.89E-04
MF	GO:0042379	chemokine receptor binding	2/7	74/18496	3.27E-04	2.13E-03	6.89E-04
MF	GO:0004222	metalloendopeptidase activity	2/7	110/18496	7.22E-04	3.75E-03	1.22E-03
KEGG	hsa04061	Viral protein interaction with cytokine and cytokine receptor	2/3	100/8779	3.83E-04	3.83E-03	4.03E-04
KEGG	hsa04062	Chemokine signaling pathway	2/3	192/8779	1.41E-03	7.04E-03	7.41E-04
KEGG	hsa04060	Cytokine-cytokine receptor interaction	2/3	297/8779	3.35E-03	1.12E-02	1.17E-03
KEGG	hsa05330	Allograft rejection	1/3	38/8779	1.29E-02	2.44E-02	2.57E-03
KEGG	hsa05332	Graft-versus-host disease	1/3	42/8779	1.43E-02	2.44E-02	2.57E-03

GO, Gene Ontology; KEGG, Kyoto Encyclopedia of Genes and Genomes; BP, Biological Process; CC, Cellular Component; MF, Molecular Function; MRDEGs, Macrophage-Related Differentially Expressed Genes.

### 3.2 Unsupervised clustering identifies two intrinsic immune subtypes with distinct clinicogenomic features

To assess whether the macrophage-related hub genes were associated with distinct overall immune microenvironment backgrounds, and to provide a framework for interpreting the immune context of the subsequent risk groups, we performed consensus clustering on the TCGA-COADREAD cohort using the infiltration profiles of 28 immune cell types. This analysis robustly partitioned all samples into two distinct subtypes, designated as Immune Cluster 1 (C1) and Immune Cluster 2 (C2). **Figure 2A** shows the consensus matrix heatmap at k=2, illustrating the stable clustering of samples into two subtypes. **Figure 2B** displays the cumulative distribution function (CDF) curves for different k values, and **Figure 2C** shows the delta area plot of the CDF curves, supporting k=2 as an optimal clustering number. Comparative analysis revealed substantial disparities in the abundances of various immune cell types between the two clusters (**Figure 2D**), providing a characterization of the overall immune context associated with the macrophage-related gene expression. We further investigated the association of these intrinsic immune subtypes with key genomic and tumor microenvironment features. While no significant difference in MSI status was observed (**Figure 2E**, left panel), C2 exhibited a significantly higher TMB than C1 (**Figure 2E**, right panel). Additionally, the TIDE score, a predictor of immunotherapy response, was significantly elevated in Cluster 2 (**Figure 2F**). These findings delineate two fundamentally distinct tumor microenvironments in CRC, characterized by divergent immune composition, genomic features, and immunosuppressive potential, providing a context for interpreting the macrophage-related prognostic signature.

**Figure 2:**
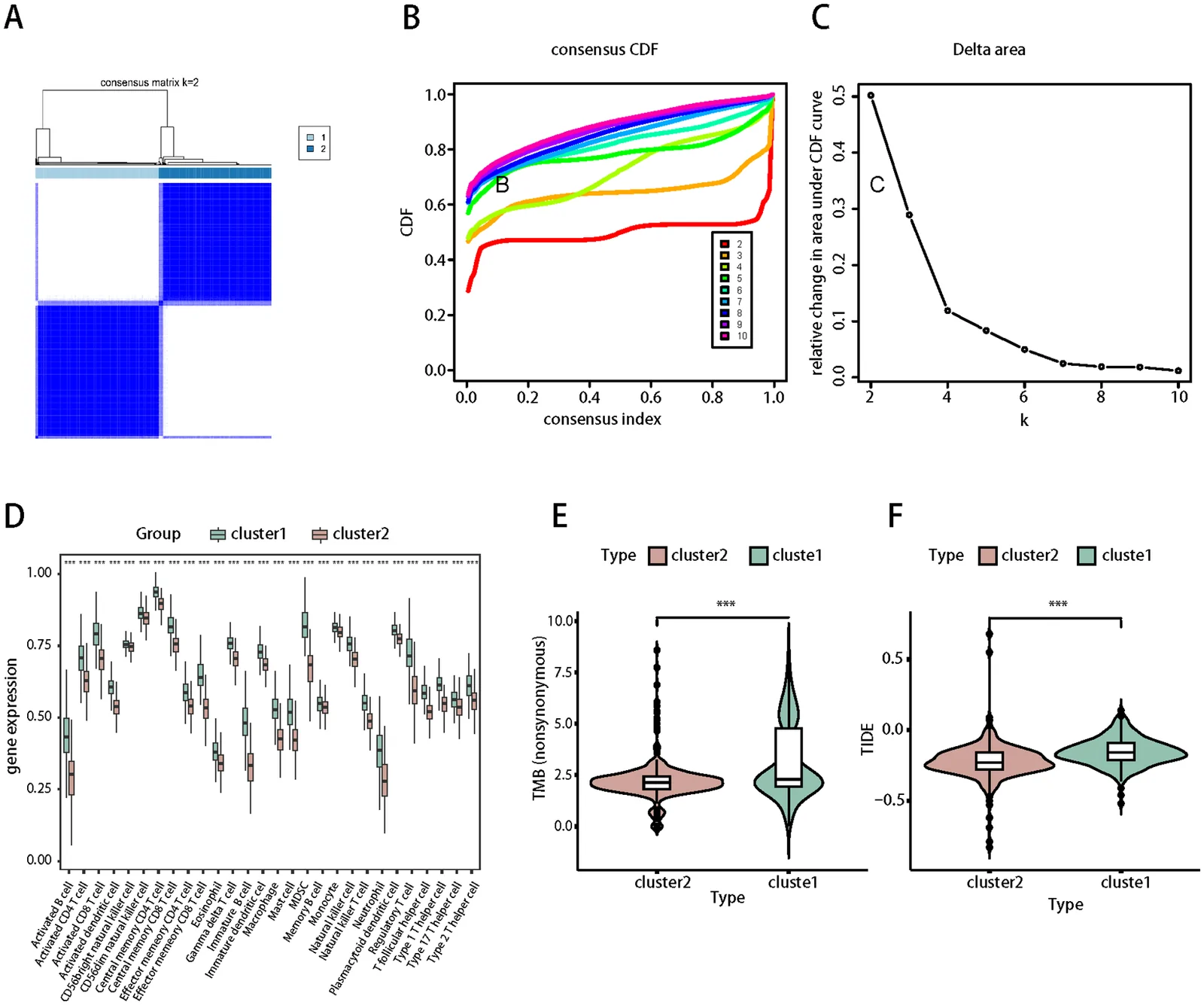
Identification and characterization of two intrinsic immune subtypes in CRC. **(A)** Consensus matrix heatmap from consensus clustering (*k* = 2), indicating stable sample partitioning into two immune subtypes (C1 and C2). **(B)** Cumulative distribution function (CDF) curves for different k values (k=2 to 10). **(C)** Delta area plot of the CDF curves, supporting k=2 as the optimal clustering number. **(D)** Heatmap showing the abundance of immune cells in Immune Cluster C1 and C2. Asterisks indicate statistical significance based on nominal P values. The text reports results based on FDR-adjusted *P* values (Benjamini-Hochberg method). (*p < 0.05, **p < 0.01, ***p < 0.001). **(E)** Comparison of tumor mutational burden (TMB) between C1 and C2. TMB is significantly higher in C2 than in C1 (***p < 0.001). **(F)** Comparison of Tumor Immune Dysfunction and Exclusion (TIDE) scores between C1 and C2. No significant difference was observed (N.S., p > 0.05).

### 3.3 Construction of a prognostic signature based on macrophage-related hub genes

To select the final signature genes for prognostic modeling from the eight hub MRDEGs identified in the previous section, we performed LASSO-Cox regression analysis. **Figure 3A** shows the LASSO coefficient profiles of the 8 hub MRDEGs across different lambda values, where candidate genes were progressively shrunk towards zero with increasing penalty. **Figure 3B** displays the 10-fold cross-validation results used to determine the optimal lambda, ultimately retaining *CCL24* and *MMP12* as the two signature genes for constructing the risk model. The risk score was calculated as follows: RiskScore=(−0.048*CCL24)+(−0.025*MMP12). The coefficients for both *CCL24* and *MMP12* are negative, indicating that higher expression of these genes corresponds to a lower risk score, while lower expression corresponds to a higher risk score. The larger absolute coefficient for *CCL24* indicates that its expression contributes more heavily to the final risk score than *MMP12*. Importantly, both *CCL24* and *MMP12* are derived from the macrophage-related hub genes, and their expression patterns are linked to macrophage-associated biological processes, including immune regulation and tissue remodeling, as identified in our single-cell analysis. Patients were categorized into a high-risk group and a low-risk group based on the median value of the risk score. The risk factor plot (**Figure 3C**) illustrates the relationship between ascending risk scores, increased mortality, and the expression patterns of the two signature genes. Since the coefficients for both *CCL24* and *MMP12* are negative, higher expression of these genes corresponds to a lower risk score, and such patients are predominantly distributed in the low-risk group.

**Figure 3:**
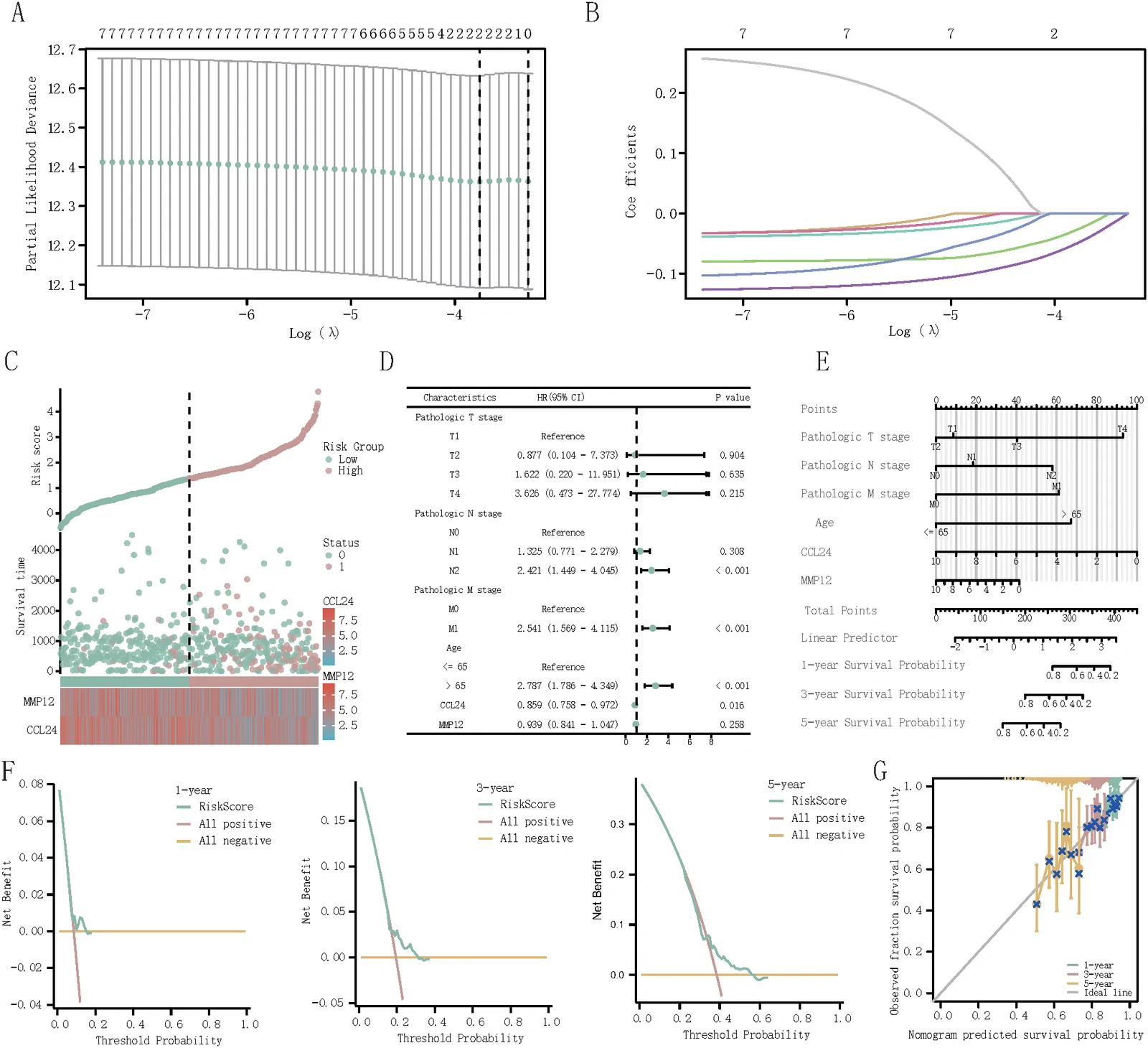
Construction and evaluation of a candidate macrophage-related prognostic signature. **(A)** LASSO coefficient profiles of the eight hub MRDEGs showing the shrinkage of coefficients with increasing lambda(λ). The vertical dashed line indicates the optimal lambda (λ) value selected for feature selection. **(B)** Ten-fold cross-validation for tuning parameter selection in the LASSO Cox regression model. The left vertical dashed line (lambda.min) indicates the λ value yielding the minimum mean cross-validated error, used to derive the final two-gene signature. **(C)** Risk factor distribution plot. Samples were arranged in ascending order of risk score and stratified into low-risk and high-risk groups based on the median risk score. The top scatter plot displays the risk score of each sample, the middle scatter plot shows survival time and survival status, and the bottom heatmap illustrates the relative expression levels of *CCL24* and *MMP12*. Since the regression coefficients of both *CCL24* and *MMP12* in the model are negative, their higher expression is predominantly distributed in the low-risk group and corresponds to a lower risk score. **(D)** Forest plots from univariate and multivariate Cox regression analyses showing the associations of clinical variables, *CCL24* and *MMP12* with overall survival. **(E)** Prognostic nomogram incorporating *CCL24*, *MMP12*, and independent clinical factors (age, N stage, M stage) to predict 1-, 3-, and 5-year survival probability. The wide “Points” range for *CCL24* highlights its substantial contribution. **(F)** The 1-year, 3-year, and 5-year decision curve analysis (DCA) plots of the CRC prognostic risk model. **(G)** Calibration Curve of CRC Prognostic Risk Model at 1, 3, and 5 Years.

Multivariate Cox regression analysis (**Table 6**) was performed to evaluate the association between the risk score, clinical variables, and overall survival. Cox regression analyses showed that *CCL24* retained an independent association with overall survival after adjustment for clinical variables, whereas *MMP12* showed a consistent protective trend but did not reach statistical significance in multivariate analysis, as shown in the forest plots (**Figure 3D**).

**Table 6:** Results of univariate and multivariable cox regression analysis.

Characteristics	Total (N)	Univariate analysis		Multivariate analysis	
		Hazard ratio (95% CI)	P value	Hazard ratio (95% CI)	P value
Pathologic T stage	640				
T1	20	Reference		Reference	
T2	111	1.000 (0.216 - 4.639)	1.000	0.877 (0.104 - 7.373)	0.904
T3	435	2.047 (0.504 - 8.317)	0.316	1.622 (0.220 - 11.951)	0.635
T4	74	6.151 (1.458 - 25.953)	0.013	3.626 (0.473 - 27.774)	0.215
Pathologic N stage	639				
N0	367	Reference		Reference	
N1	153	1.774 (1.131 - 2.781)	0.013	1.325 (0.771 - 2.279)	0.308
N2	119	3.873 (2.588 - 5.796)	< 0.001	2.421 (1.449 - 4.045)	< 0.001
Pathologic M stage	563				
M0	474	Reference		Reference	
M1	89	3.989 (2.684 - 5.929)	< 0.001	2.541 (1.569 - 4.115)	< 0.001
Gender	643				
Female	301	Reference			
Male	342	1.054 (0.744 - 1.491)	0.769		
Age	643				
<= 65	276	Reference		Reference	
> 65	367	1.939 (1.320 - 2.849)	< 0.001	2.787 (1.786 - 4.349)	< 0.001
CCL24	643	0.893 (0.799 - 0.997)	0.044	0.859 (0.758 - 0.972)	0.016
MMP12	643	0.923 (0.833 - 1.022)	0.125	0.939 (0.841 - 1.047)	0.258

Importantly, when translated into a candidate nomogram (**Figure 3E**), the “Points” scale for *CCL24* spans the widest range among all included factors. The nomogram revealed that *CCL24* contributed substantially to the total points, suggesting it may be a relatively important molecular variable in the model. To assess the predictive accuracy of our prognostic model, we performed decision curve analysis (DCA) (**Figure 3F**) and calibration analysis (**Figure 3G**) for 1-, 3-, and 5-year overall survival. Both analyses indicated that the model demonstrated a consistent net benefit and favorable calibration for 5-year survival predictions in this cohort, followed by 3-year and 1-year predictions.

### 3.4 Biological characterization and therapeutic prediction of the prognostic risk groups

To further characterize the tumor immune microenvironment context associated with the macrophage-related prognostic signature, and to evaluate whether the risk groups defined by this signature correspond to distinct immune states, we compared the immune cell infiltration landscape between the high-risk and low-risk groups. After FDR correction, significant differences in the abundance of macrophage subtypes were observed, including M0 and M2 macrophages, as well as CD8+ T cells (adjusted *P* < 0.05). The remaining cell types showed nominal differences that did not survive FDR correction (**Figure 4A**). To further investigate the therapeutic implications of the risk stratification, we used pRRophetic to estimate the predicted IC50 of common chemotherapeutic and targeted drugs based on the GDSC database (28). Notably, the high-risk group displayed a predicted lower sensitivity to sorafenib (**Figure 4B**), suggesting transcriptomic differences between risk groups that may be associated with drug response, but experimental validation is required. Furthermore, we observed differential expression of multiple immune checkpoint genes between the high- and low-risk groups (**Figure 4C**), which may indicate differences in immune checkpoint expression patterns, rather than indicating differential responses to immune checkpoint blockade therapy.

**Figure 4:**
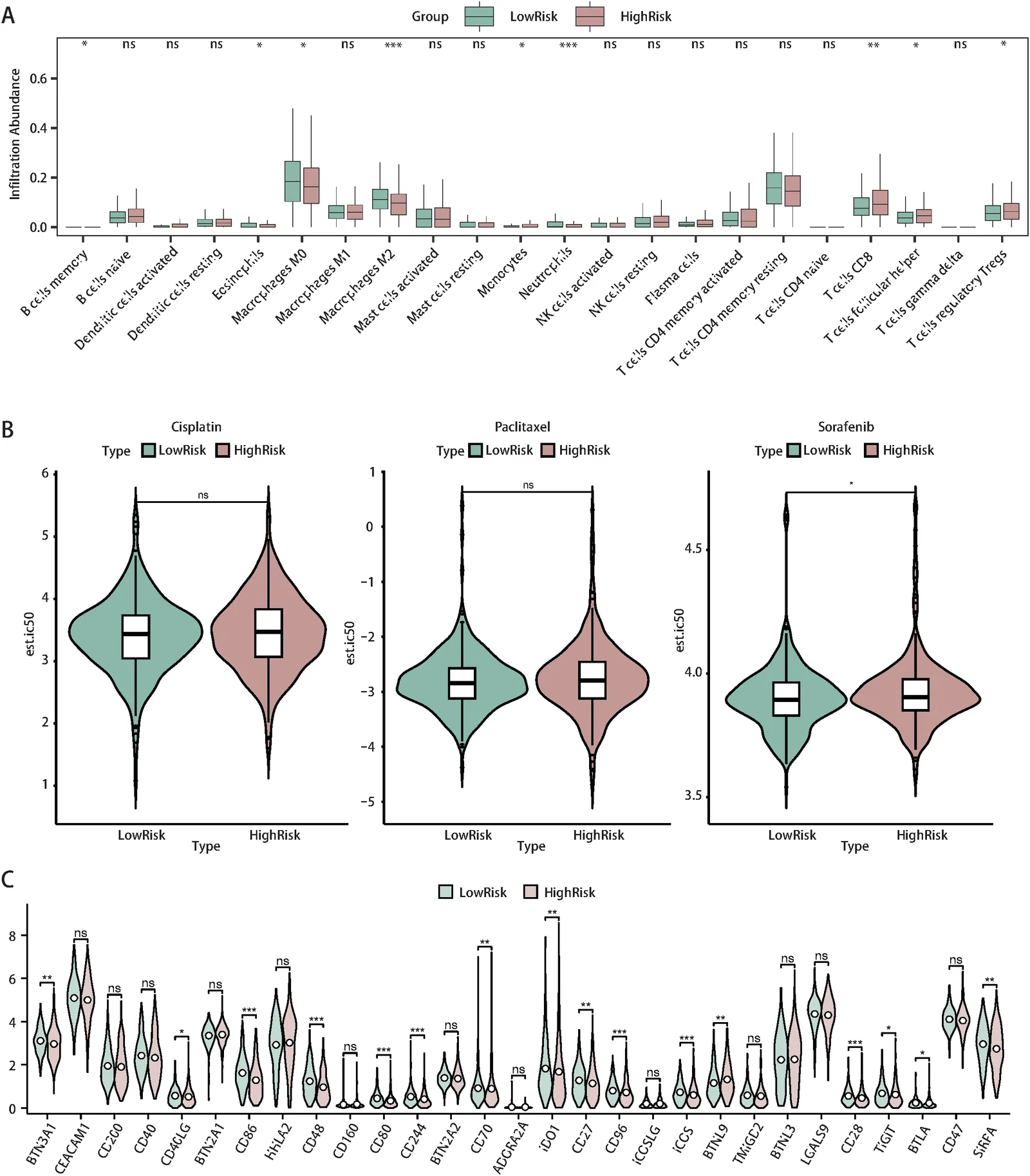
Biological and clinical characterization of the prognostic risk groups. **(A)** Box plots comparing the infiltration levels of 22 immune cell types between the high-risk and low-risk groups. Asterisks indicate nominal P values. The text reports results based on FDR-adjusted P values (Benjamini-Hochberg method) (*p < 0.05, **p < 0.01, ***p < 0.001). **(B)** Box plots comparing the predicted sensitivity (log-transformed IC50 values) to three chemotherapeutic/targeted agents between the high-risk and low-risk groups. A lower IC50 indicates higher predicted drug sensitivity. Sorafenib sensitivity is highlighted (N.S., not significant, *p < 0.05). **(C)** Heatmap showing the differential expression of key immune checkpoint genes between the high-risk and low-risk groups.

### 3.5 Experimental validation and mechanistic insights from cell-cell communication analysis

To validate the key findings, we first assessed the mRNA expression of the eight hub MRDEGs in CRC clinical specimens. qRT-PCR was used to assess the mRNA expression patterns of the eight hub MRDEGs (*CCL24*, *ADAMDEC1*, *CHI3L1*, *GZMB*, *CCL23*, *CD163L1*, *MMP12*, and *CDKN3*) in paired CRC and adjacent normal tissues. The results showed that *CCL24*, *CHI3L1*, *GZMB*, *MMP12*, and *CDKN3* were significantly upregulated in CRC tissues compared to adjacent normal tissues, whereas *ADAMDEC1* was significantly downregulated (**Figure 5A**). Having established the mRNA expression patterns of all eight hub genes, we next focused on validating the protein expression of the two genes (*CCL24* and *MMP12*) that constituted the final prognostic signature derived from LASSO-Cox regression analysis. Western blot analysis revealed concordant upregulation of both CCL24 and MMP12 at the protein level in tumor tissues (**Figures 5B, C**), which is consistent with our prior computational predictions and qRT-PCR results.

**Figure 5:**
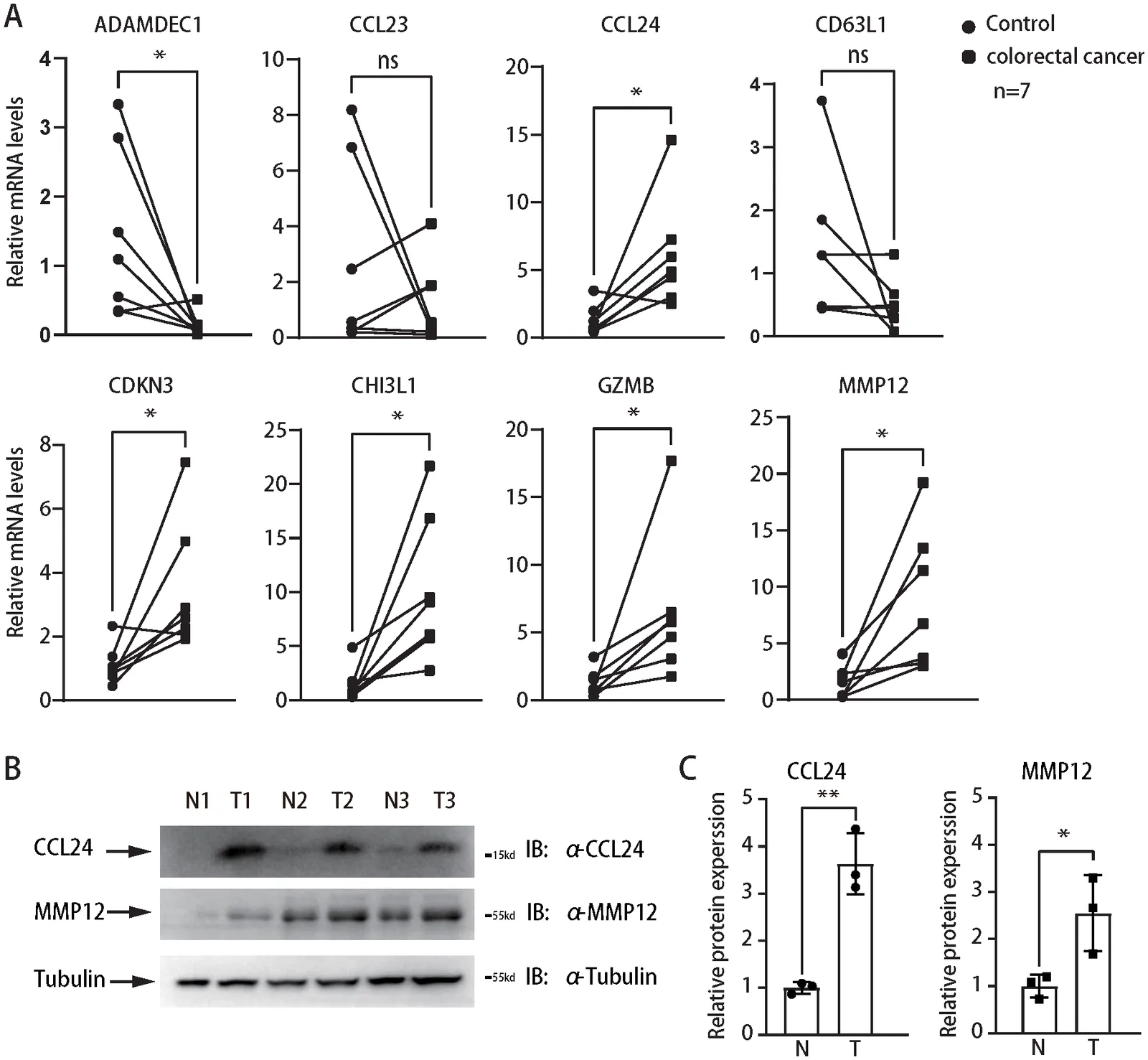
Experimental validation of hub gene expression in independent clinical samples. **(A)** qRT-PCR analysis of the eight hub MRDEGs in paired CRC tumor and adjacent normal tissues (n = 7 pairs). Statistical significance was assessed using the paired t-test. *p < 0.05, **p < 0.01; ns, not statistically significant. **(B, C)** Western blot analysis of CCL24 and MMP12 protein expression in paired CRC tumor (T) and adjacent normal (N) tissues (N = 3 pairs) **(B)** and the corresponding statistical results **(C)**. Tubulin was used as the loading control. Molecular weight markers are indicated. Statistical significance was assessed using the paired t-test. *p < 0.05, **p < 0.01.

To explore the potential mechanisms through which macrophage-related genes influence patient prognosis, we interrogated the cell-cell communication network within the scRNA-seq data using CellPhoneDB. This analysis revealed extensive interaction networks between macrophages and other cell types, including epithelial cells and T cells (**Figure 6A**). A notably strong interaction signal was identified between HLA-DRA (expressed by macrophages) and CD4 (expressed by CD4^+^ T cells) (**Figure 6B**). This finding suggests that macrophages may regulate adaptive immune responses via antigen presentation, suggesting a potential mechanistic link to the observed prognostic associations. Detailed results from SCENIC and pseudotime trajectory analyses for the macrophage subtypes are provided in **Supplementary Figures 8**, **S9**, respectively.

**Figure 6:**
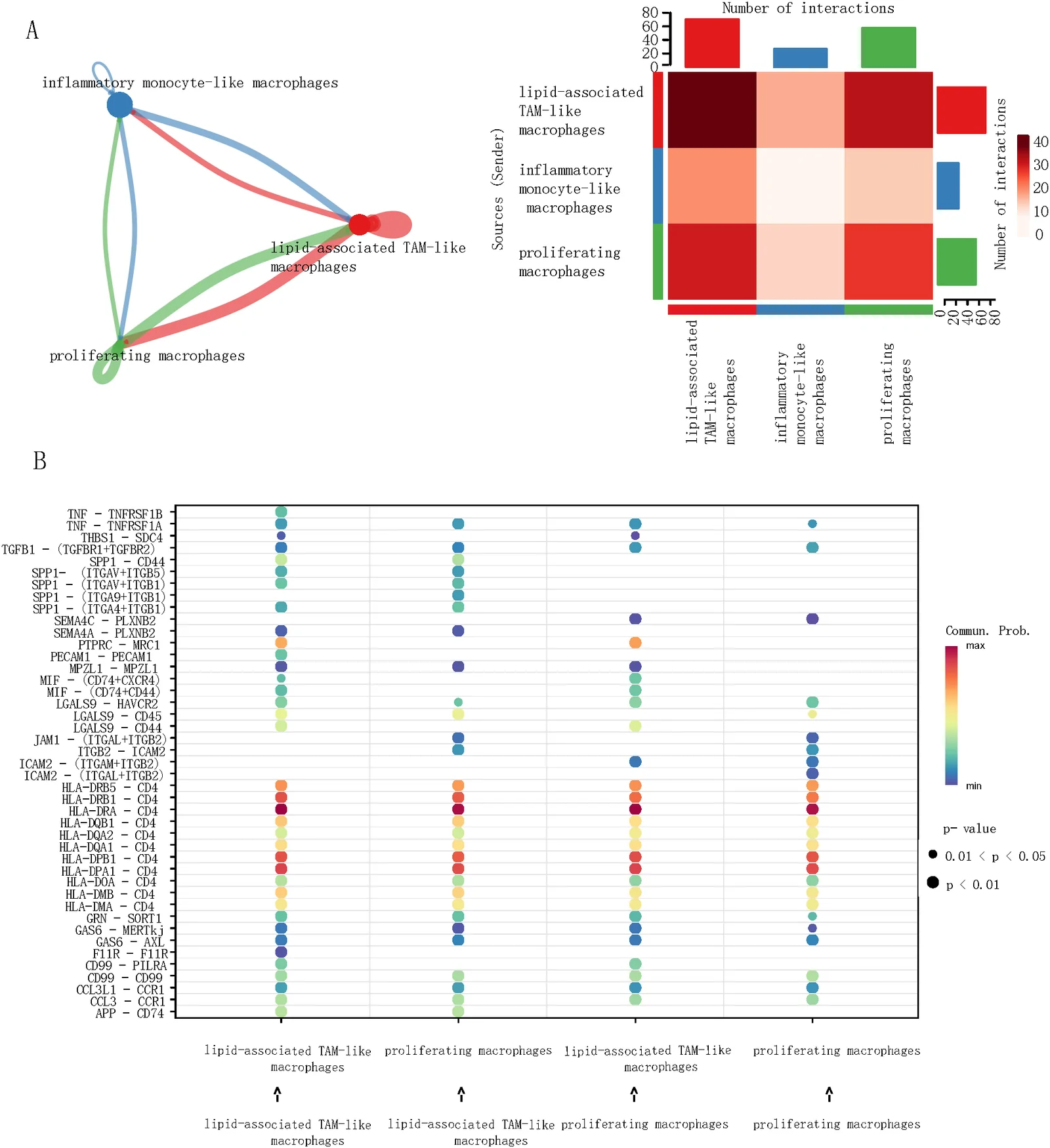
Cell-cell communication analysis reveals potential immune-regulatory mechanisms. **(A)** Network diagram and heatmap summarizing the significant ligand-receptor interactions between the lipid-associated TAM-like macrophages and other major cell types in the CRC tumor microenvironment. **(B)** Dot plot highlighting the specific and strong interaction between *HLA-DRA* (expressed by macrophages) and *CD4* (expressed on CD4^+^ T cells), as identified by CellPhoneDB analysis.

## 4 Discussion

The tumor microenvironment (TME) of CRC, particularly its highly heterogeneous immune cell communities, is a critical determinant of disease progression and treatment response (29). In this study, we integrated single-cell RNA sequencing data with multi-cohort bulk transcriptomic datasets to systematically delineate the heterogeneity of myeloid immune cells, especially macrophages, in CRC. This single-cell analysis revealed distinct macrophage subtypes. Through differential expression analysis and intersection with both predefined macrophage-related genes and bulk transcriptomic cohorts, we prioritized 56 macrophage-related DEGs (MRDEGs) and subsequently 8 hub MRDEGs. From these, a two-gene prognostic signature (*CCL24* and *MMP12*) was constructed via LASSO-Cox regression. The primary contribution of this study is to bridge the gap between CRC macrophage subtype heterogeneity at the single-cell level and prognostic information from bulk cohorts, thereby positioning *CCL24* and *MMP12* as candidate prognostic molecules with a well-defined macrophage-related background. This signature not only demonstrated prognostic value but also revealed distinct immune context and predicted drug sensitivity profiles between risk groups, suggesting its potential relevance to the tumor immune microenvironment.

Our single-cell analysis identified three functionally distinct macrophage subpopulations. Notably, a macrophage subset characterized by higher *NR1H3* (LXRα) expression and enriched regulon activity was observed. *NR1H3*, a key regulator of cholesterol metabolism and inflammatory responses widely studied in conditions like atherosclerosis (30), regulates the expression of *Arg*1 and *IRF8* in macrophages (31). The potential role of this *NR1H3*/LXRα regulon in a specific TAM subtype is highlighted here, suggesting it may contribute to lipid-associated metabolic reprogramming within the TAM population (32, 33). This observation offers a potential clue for targeting TAM metabolism to modulate anti-tumor immunity (34), although further functional validation is required. This contrasts with the conventional view that simplifies TAMs into a binary M1/M2 polarization paradigm (35), as accumulating evidence points to more complex functional states consistent with our findings (36).

Our single-cell analysis not only identified functionally distinct macrophage subpopulations but also revealed complex associations within the CRC immune landscape. For instance, immune subtypes defined by cellular composition (e.g., C1 vs. C2) exhibited significant differences in TMB and immune escape potential (TIDE score). This finding underscores the complexity of tumor immunology, suggesting that distinct cellular ecosystems may coexist with unique genomic alterations and immune editing states, which may help explain why some patients with high TMB remain refractory to immunotherapy (37).Through multi-tiered data cross-screening, we pinpointed eight macrophage-related genes that exhibited stable differential expression across both the cellular subtype and bulk tissue levels. Among these, *MMP12* and *CCL24* emerged as the core components of our prognostic risk model. *MMP12*, a macrophage-derived elastase, is known to degrade the extracellular matrix and promote tumor invasion, with its high expression associated with poor prognosis in various cancers (38). Our work not only confirms this but also crucially anchors its expression to specific macrophage subpopulations using single-cell data.

Similarly, *CCL24* (eotaxin-2) has been primarily studied in allergic diseases and eosinophil recruitment (39), with some studies suggesting a pro-tumorigenic role in cancers such as renal cell carcinoma (40) and a recent report linking it to liver metastasis (41). Our work extends this evidence by explicitly associating *CCL24* with macrophage subtypes and validating its high expression in CRC tissues using clinical samples, thereby characterizing its expression in primary CRC lesions for the first time.

Interestingly, the risk model indicates that the high-risk group (corresponding to a higher risk score) is associated with low expression of both *CCL24* and *MMP12*. Enrichment analysis further showed significant enrichment of “eosinophil chemotaxis” and “Th2-type immune response” pathways in the high-risk group. This seemingly paradoxical observation may reflect a specific tumor immune microenvironment context, rather than indicating that low expression of these genes directly drives poor prognosis (42). Specifically, the low expression of *CCL24*/*MMP12* might signify an immunosuppressive state characterized by robust recruitment of eosinophils and induction of Th2 polarization via alternative pathways (43) (e.g., *CCL11*, *CCL26*), which are strongly linked to poor prognosis (44). This provides a fresh perspective on immune evasion in CRC that extends beyond the level of a single chemokine. It should also be noted that differential expression analysis between tumor and normal tissues (e.g., upregulation of *CCL24*/*MMP12* in CRC) and patient survival stratification are two distinct analytical levels and should not be directly equated.

Although numerous transcriptome-based prognostic markers for CRC exist, many models lack in-depth interpretation of their cellular origins and functions (45). The strength of our study lies in deriving model genes directly from single-cell analysis, thereby providing clear biological interpretability. For example, the core genes *CCL24* and *MMP12* are explicitly localized to specific macrophage subsets and are closely linked to immunosuppressive and chemotactic pathways.

The risk score was associated with survival and multidimensional clinical features. In terms of immune characteristics, elevated risk scores positively correlated with increased abundance of immunosuppressive cell types (including M2 macrophages and Tregs) and a potentially exhausted state of cytotoxic T cells. This aligns with the upregulation of immune checkpoints like IDO1 and TIGIT in the high-risk group, collectively depicting an immunologically “cold” tumor or an immunosuppressive microenvironment. Regarding therapeutic implications: pRRophetic-based prediction suggested that the high-risk group may have a higher estimated IC50 for Sorafenib; this result requires drug sensitivity experimental validation.

Several prognostic models for CRC exist based on bulk transcriptome data (46–49). Unlike models that may be composed of genes with unclear cellular origins, our model explicitly links the prognostic genes (*CCL24* and *MMP12*) to specific macrophage subtypes, as identified through our single-cell analysis. Therefore, this model may provide a complementary macrophage-related biological context to existing prognostic tools, rather than claiming superiority over them. The true predictive performance of this model relative to established ones would require a direct head-to-head comparison in a unified cohort, which is beyond the scope of the current study. Compared to many published models, our two-gene signature offers simplicity, and when combined with classic TNM staging, our candidate nomogram provided an estimation of individualized survival probability in the current cohort. Furthermore, decision curve analysis (DCA) suggested a positive net benefit at 1, 3, and 5 years in this cohort, indicating that this model may merit further validation. This study has several limitations. First, the conclusions derived from the single-cell data, which were based on a limited sample size, require validation in larger and more diverse populations. Second, the prognostic model was constructed using retrospective data and has not been prospectively validated in independent clinical cohorts. Moreover, we did not perform a direct head-to-head comparison between our signature and established CRC prognostic tools (e.g., Oncotype DX, ColoPrint), which differ in detection platforms, target populations, and endpoint definitions. Future studies should systematically evaluate the predictive performance of different models using standardized platforms and prospective cohorts (50). Third, the functional characterization of macrophage subtypes—including differential marker expression, GSVA pathway analysis, SCENIC regulon inference, and pseudotime trajectory analysis—is primarily computational and hypothesis-generating, requiring validation through flow cytometry, spatial transcriptomics, or functional assays (6). Additionally, the drug sensitivity predictions (based on the pRRophetic algorithm and GDSC database), the immune checkpoint expression differences between risk groups, and the prognostic nomogram (built solely on the TCGA cohort) are all derived from computational analyses or retrospective data and lack independent external or prospective validation. Therefore, all findings presented in this study should be considered hypothesis-generating and warrant further investigation.

## 5 Conclusion

In summary, through integrated multi-omics analysis, this study provides additional resolution beyond conventional cell classifications by revealing distinct transcriptional states of macrophages and their potential functional differences, providing new insights into CRC biology. It is the first to establish *CCL24* as a key molecule associated with CRC prognosis, potentially functioning through the modulation of eosinophil-related pathways. The concise prognostic model, constructed based on *CCL24* and *MMP12*, integrates features of the tumor immune microenvironment, genomic instability, and potential therapeutic response. This study provides a candidate prognostic stratification model that requires further validation in independent cohorts and prospective studies.

## Data Availability

The original contributions presented in the study are included in the article/**Supplementary Material**. Further inquiries can be directed to the corresponding authors.

## Supplementary material

Supplementary Figure 1 UMAP feature plots showing representative marker-gene expression patterns for the major cell types identified in **Figure 1A**. Marker genes were selected based on the single-cell differential marker analysis of each major cell type.

Supplementary Figure 2 **(A)** UMAP plot and violin plots showing macrophage subtypes and representative marker-gene expression: Cluster 0/CLC, inflammatory monocyte-like macrophages; Cluster 1/ C2orf48, lipid-associated TAM-like macrophages; Cluster 2/ HS3ST2, proliferating macrophages. The top five differentially expressed marker genes for each subtype are visualized as UMAP feature plots in **Supplementary Figure 3**. **(B)** Heatmap of GSVA scores for hallmark pathways in the three macrophage subtypes, highlighting differential activities in pathways like inflammatory response and ribosome assembly. The color scale bar represents the standardized GSVA score (or pathway activity), with blue indicating lower pathway activity and red indicating higher pathway activity.

Supplementary Figure 3 UMAP feature plots showing the top five differentially expressed marker genes for each macrophage subtype shown in **Supplementary Figure 2A**. The marker genes for each macrophage subtype were derived from single-cell differential expression analysis comparing the three macrophage subtypes.

Supplementary Figure 4 Epithelial cell subtyping. **(A)** UMAP plot showing the sub clustering of epithelial cells into distinct subtypes. **(B)** Violin plots showing the expression of canonical marker genes for each epithelial subtype.

Supplementary Figure 5 Fibroblast cell subtyping. **(A)** UMAP plot showing the subclustering of fibroblasts into distinct subtypes. **(B)** Violin plots showing the expression of canonical marker genes for each fibroblast subtype.

Supplementary Figure 6 Validation of hub gene expression in bulk cohorts. Heatmaps showing the consistent differential expression patterns of the eight hub genes between tumor and normal tissues across the five validation bulk RNA-seq cohorts (TCGA-COADREAD, GSE20916, GSE73360, GSE44861 and GSE74602).

Supplementary Figure 7 Genomic alteration landscape of the eight hub MRDEGs. **(A, B)** The lollipop plot and mutation frequency histogram of copy number variation (CNV) in the TCGA-COADREAD dataset for MRDEGs with CNV occurrence. **(C)** Somatic mutations of MRDEGs in CRC samples from the TCGA-COADREAD.

Supplementary Figure 8 SCENIC analysis of macrophage subtypes. **(A)** Regulon activity heatmap illustrating the transcription factor regulon activities specific to the three macrophage subtypes (inflammatory monocyte-like macrophages, lipid-associated TAM-like macrophages, and proliferating macrophages) (red represents high regulation intensity, blue represents low regulation intensity). **(B)** Regulate intensity UMAP illustrating the transcription factors NR1H3_extended (53 g) and NR1H3 (33 g), which are the strongest regulators of Macrophage cell subtypes.

Supplementary Figure 9 Pseudotime trajectory analysis reveals the differentiation dynamics of macrophage subpopulations. **(A)** Cell differentiation pseudotime plot. **(B)** Developmental trajectory of all cells. **(C)** Expression heatmap of 56 MRDEGs along the pseudotime axis.
