# Correction: Integrated single-cell and bulk tissue analyses reveal distinct macrophage subtypes and a candidate prognostic signature in colorectal cancer: implications for tumor immune characterization

**DOI:** 10.3389/fimmu.2026.2032247

**Published:** 2026-10-26

**Authors:** Hui Chen, Zhipeng Li, Zhen Lin, Lijun Wan, Yuhang Gong, Zhibin Lv, Jinfeng Hu, Dun Pan

**Affiliations:** 1 Department of Gastrointestinal Surgery, First Affiliated Hospital of Fujian Medical University, Fuzhou, China; 2 Fujian Research Institute of Abdominal Surgery, Fuzhou, China; 3 First Affiliated Hospital of Fujian Medical University Binhai Campus, Fuzhou, China; 4 Fujian Medical University School of Basic Medical Sciences, Fuzhou, China

**Keywords:** CCL24, colorectal cancer, macrophage, MMP12, prognosis, single-cell RNA sequencing, tumor immunology

Author “Hui Chen, Zhipeng Li” was erroneously omitted as equal contributing author. We have now added for this author: “†These authors have contributed equally to this work and share first authorship”.

The original version of this article has been updated.

